# Reduced blood brain barrier breakdown in P-selectin deficient mice following transient ischemic stroke: a future therapeutic target for treatment of stroke

**DOI:** 10.1186/1471-2202-11-12

**Published:** 2010-02-02

**Authors:** Albert Y Jin, Ursula I Tuor, David Rushforth, Jaspreet Kaur, Robert N Muller, Jodie Lee Petterson, Sébastien Boutry, Philip A Barber

**Affiliations:** 1 Department of Clinical Neurosciences, University of Calgary, Calgary, Alberta, Canada; 2 National Research Council of Canada, Institute for Biodiagnostics (West), Calgary, Alberta, Canada; 3 Department of General, Organic and Biomedical Chemistry, NMR and Molecular Imaging Laboratory, Université de Mons-Hainaut, Mons, Belgium

## Abstract

**Background:**

The link between early blood- brain barrier (BBB) breakdown and endothelial cell activation in acute stroke remain poorly defined. We hypothesized that P-selectin, a mediator of the early phase of leukocyte recruitment in acute ischemia is also a major contributor to early BBB dysfunction following stroke. This was investigated by examining the relationship between BBB alterations following transient ischemic stroke and expression of cellular adhesion molecule P-selectin using a combination of magnetic resonance molecular imaging (MRMI), intravital microscopy and immunohistochemistry. MRMI was performed using the contrast, gadolinium diethylenetriaminepentaacetic acid (Gd-DTPA) conjugated to Sialyl Lewis X (Sle^x^) where the latter is known to bind to activated endothelium via E- or P selectins. Middle cerebral artery occlusion was induced in male C57/BL 6 wild-type (WT) mice and P-selectin-knockout (KO) mice. At 24 hours following middle cerebral artery occlusion, T_1 _maps were acquired prior to and following contrast injection. In addition to measuring P- and E-selectin expression in brain homogenates, alterations in BBB function were determined immunohistochemically by assessing the extravasation of immunoglobulin G (IgG) or staining for polymorphonuclear (PMN) leukocytes. *In vivo *assessment of BBB dysfunction was also investigated optically using intravital microscopy of the pial circulation following the injection of Fluorescein Isothiocyanate (FITC)-dextran (MW 2000 kDa).

**Results:**

MRI confirmed similar infarct sizes and T_1 _values at 24 hours following stroke for both WT and KO animals. However, the blood to brain transfer constant for Gd DTPA (K_gd_) demonstrated greater tissue extravasation of Gd DTPA in WT animals than KO mice (P < 0.03). In the P selectin KO mice, Δ T_1 _stroke -Δ T_1 _contralateral control cortex, decreased significantly in the Gd-DTPA(sLe^X^) group compared to Gd-DTPA, indicative of sLe^X ^mediated accumulation of the targeted contrast agent. Regarding BBB function, in the P-selectin KO mice compared to WT control mice, there was an attenuation in the extravasation of IgG (P < 0.001), a trend for decreased FITC extravasation and less infiltration of PMN leukocytes (P < 0.001) thereby supporting the observed increase in K_gd _permeability in stroke brain of WT compared to KO mice.

**Conclusion:**

P-selectin expression contributes to enhanced BBB dysfunction at 24 hours after transient focal cerebral ischemia.

## Background

Leukocyte recruitment occurs after ischemia/reperfusion resulting in local tissue damage and compromised microvascular perfusion. Central to this process is the vascular endothelium expression of P- and E-selectin in the first few minutes to hours after cerebral ischemia, leading to polymorphonuclear (PMN) leukocyte or neutrophil migration into brain tissue, cytokine release and free radical-mediated damage [1]. During this time, blood brain barrier (BBB) injury can lead to an increase in vascular permeability and brain edema, exacerbating the initial ischemic injury [2]. Although neuroinflammatory processes contribute to BBB breakdown in many conditions [3], the link between early BBB dysfunction and endothelial cell activation in acute stroke is unclear. There is evidence that the inhibition of selectin adhesion molecules reduces brain injury and inhibits neutrophil and platelet accumulation after focal ischemia in mice [4,5], but the mechanisms involved in this protection remain speculative [6].

We hypothesized that since there is evidence that P-selectin is a mediator of the early phase of leukocyte recruitment in acute ischemia it is also a major contributor to enhancing the early BBB dysfunction following stroke. Thus, we compared alterations in BBB permeability 24 hours following transient focal cerebral ischemia in wild type (WT) control mice to those in P-selectin knockout (KO) mice. The results demonstrate that following stroke P-selectin expression contributes to BBB injury as detected using a combination of magnetic resonance molecular imaging (MRMI), intravital microscopy and immunohistochemical methods. MRMI was performed using gadolinium (Gd) diethylenetriaminepentaacetic acid (DTPA) conjugated to a Sialyl Lewis X (Slex) - a mimetic of P-selectin glycoprotein ligand-1 tetrasaccharide sialyl Lewis X which mediates the binding of leukocytes and platelets to activated endothelium [7,8].

## Methods

### Animal Preparation

All animal procedures were approved by the Animal Care Committees at the University of Calgary and the National Research Council of Canada. Transient middle cerebral artery occlusion of 30 minutes duration was induced in thirty four 30-35 gram male C57/BL 6 wild-type mice or 29 P-selectin-deficient or knockout mice (Jackson Laboratory) using the intraluminal suture occlusion method as described previously [9]. Control animals with sham surgery included all surgical steps except for middle cerebral artery occlusion (n = 8). Temperature regulation during ischemia and reperfusion was achieved using an intra-abdominal telemetry probe (Data Sciences International) with feedback to a heating pad to remove the potentially confounding influence of hypothermia during the reperfusion period. At 24 hours, the intra-abdominal probe was removed under aseptic conditions and temperature was then regulated by feedback from a rectal temperature probe during any subsequent procedures.

### Magnetic Resonance Imaging

At 24 hours after stroke, animals were anesthetised with isoflurane for magnetic resonance (MR) imaging using a 9.4 T MR system equipped with a Bruker Avance console as described previously [9]. Briefly, T_2 _scans were acquired using a spin-echo sequence with a 2 × 2 cm^2 ^field of view, a 256 × 128 data matrix, a repetition time of 2750 ms and 24 echoes with 10 msec echo-spacing for 8 slices 1 mm thick. T_1 _scans were acquired using a Contrast FAST sequence with a 2.5 × 2.5 cm^2 ^field of view, a 128 × 128 data matrix, a repetition time of 3.5 ms, an echo time of 2 msec, a flip angle of 12° and 20 inversion time points for one 1 mm thick slice through the infarct. T_1 _and T_2 _maps were determined using local imaging software (Marevisi, National Research Council).

For the experimental protocol, T_1 _and T_2 _scans were first acquired prior to contrast injection. Then, either DTPA (molecular weight 938 g/mol) or targeted contrast agent using a mimetic of Sialyl Lewis^X^, coupled to Gd-DTPA (Gd-DTPA-(sLeX), molecular weight 1758 g/mol) was injected intravenously at a dose of 0.1 mmol Gd/kg body weight. Subsequently, T_1 _maps were acquired every 11 minutes for 55 minutes to allow Gd-DTPA-(sLeX) to bind to its target and to allow non-targeted (Gd-DTPA) contrast to at least partially clear from the circulation.

The first 33 minutes post injection was used to assess blood-brain barrier function by determining the transfer constant for Gd-DTPA into the brain (K_Gd_). K_Gd _was calculated using T_1 _measures in the sagittal sinus and in regions of interest using a Patlak plot similar to that described by Ewing et al [10]. The regions of interest included the parietal cortex in the infarct core and the equivalent contralateral control cortex. T_1 _maps were also used to determine targeted contrast binding by calculating the quantity Δ T_1 _Stroke - Δ T_1 _Control Hemisphere which was defined as: [Post - pre-contrast mean T_1 _value of the stroke region] - [Post - pre-contrast mean T_1 _value of a corresponding region in the contralateral cortex]. After imaging, the animals were euthanized by intracardiac injection of sodium pentobarbital (70 mg/kg).

Infarct areas and volumes were calculated using the T_2 _MR images. The infarct area in each T_2 _slice was readily apparent as a region of increased T_2_. The calculation of the infarct area included a measure of the number of pixels in the ipsilateral hemisphere (with the exception of the ventricles) having a T_2 _value greater than a threshold of 2 standard deviations above the mean T_2 _in the contralateral cortex. For each slice, the infarct area was corrected for edema (Ipsilateral infarct area (uncorrected)/((Ipsilateral hemisphere area/Contralateral hemisphere area)), and from this, the radius of this area was derived. Then the infarct and ipsilateral hemispheric volume between slices was calculated by the cone frustum volume approximation and the infarct and hemispheric tip volumes anteriorly and posteriorly were calculated by the cone volume approximation [11,12].

### Protein and Immunohistochemical Tissue Analyses

P- and E-selectin expression was measured in samples of cortical tissue homogenates from the hemispheres contralateral and ipsilateral to the infarct. These levels were quantified using commercially available enzyme-linked immunosorbent assay (ELISA) kits (Quantikine sP-selectin or sE-selectin ELISA Kit, R&D Systems). Total protein in each sample was determined by the modified Lowry assay method.

After 24 h, animals were injected with an overdose of sodium pentobarbital and perfused transcardially with 0.1 M phosphate buffered saline followed by 4% paraformaldehyde. Brains were removed, dehydrated and embedded in paraffin blocks. Serial coronal paraffin sections (10 μm) thick were cut and stained with hematoxylin and eosin for identification of infarcted tissue.

The presence of systemic immunoglobulin G (IgG) within brain was assessed immunohistochemically in 10 μm thick paraffin embedded coronal sections cut at the level of bregma +1.2 mm. Sections were stained with goat anti-mouse IgG antibody (1:200 concentration), using horseradish peroxidase-streptavidin (1:400 concentration) and diaminobenzidine for visualization. Sections from multiple treatment groups were processed together using standardized techniques for staining and measurement. Relative staining intensity was assessed using an Olympus BX 61 light microscope at 1.5× magnification. Intensity for IgG staining was quantified taking the grey levels of the darkest region in the ischemic hemisphere and subtracting it from the grey values measured in the corresponding region of darkest intensity in the contralateral hemisphere. With increasing BBB injury IgG extravasation increased resulting in darker staining and lower grey levels.

Immunohistochemical staining for polymorphonuclear (PMN) leukocytes was done on 30 μm thick cryostat sections of brain. The sections were washed in PBS initially and between all incubations. First, sections were blocked for endogenous peroxidases by incubating with a mixture of H_2_O_2 _and methanol (3:27) for 15 min. Then the sections were blocked with 10% normal goat serum for 1 h at room temperature and incubated with a mouse monoclonal antibody to granulocytes (Abcam, Cambridge, MA, USA; 1:150) overnight at 4°C. Subsequently, sections were incubated with anti-mouse biotinylated secondary antibody (Vectastain ABC Kit, Vector Labs, Burlingame, CA, USA) for 1.5 h at room temperature and incubated with Vectastain ABC reagent (Vectastain ABC kit, Vector Labs, Burlingame, CA, USA) for 30 min at room temperature. The sections were incubated with 3, 3' diaminobenzidine tetrachloride-Nickel-Cobalt (DAB-Ni-Co) substrate solution (Liquid DAB-Black Substrate Kit, Vector Labs, Burlingame, CA, USA) until color developed, dehydrated and mounted. Using an Olympus BX 61 light microscope the labelled polymorphonuclear leucocytes were identified by their positive staining and morphologically by their characteristic bilobed nucleus. The sections were assessed at ×40 magnification in five specific predefined regions which included cingulate gyrus, parietal cortex, entorhinal cortex and two striatal regions (medial and lateral) [9].

### Intravital Microscopy

Intravital microscopy was performed through a closed bone window in the anesthetized animal maintained at 36.5°C using a rectal temperature probe and heating pad. Images were acquired after tail-vein injection of FITC-dextran (MW 2000 kDa, 50 microlitres, 10 mg/mL). FITC extravasation from the pial vasculature was assessed every 5 minutes for 30 minutes with an exposure time of 20 seconds per image. Parameters for contrast, brightness and gamma correction remained unchanged throughout the measures.

### Statistical Analysis

All data were analyzed by investigators blinded to the experimental group. Data is presented as mean ± S.D. Comparisons between groups for stroke volume, mean infarct T_1 _value, K_Gd_, IgG extravasation and PMN infiltration were assessed with a one-way ANOVA. MRMI contrast effects were assessed with a two-way ANOVA with repeated measures. Animals demonstrating FITC extravasation were documented and compared using the Freeman-Halton extension of a Fisher's exact test for 2 × 3 tables. A p value of less than 0.05 was considered significant.

## Results

### Stroke injury and Selectin expression

All mice survived for 24 hours following stroke induced by transient middle cerebral artery occlusion. Cortical perfusion reductions during ischemia, measured using laser doppler flowmetry (LDF) were similar between groups with cortical perfusion decreasing to values of 14%, 17%, 19% and 16% of baseline for WT-Gd-Slex, WT-Gd, P-selectin KO- Gd-Slex and P selectin KO-Gd groups, respectively (n.s., P > 0.7, One-Way ANOVA). During reperfusion the LDF measurements were again similar between groups with values of 84%, 66%, 70% and 111% baseline flow upon reperfusion for WT-Gd-Slex, WT-Gd, P-selectin KO- Gd-Slex, and P selectin KO-Gd groups, respectively (n.s., P = 0.3, One-Way ANOVA).

**Figure 1:**
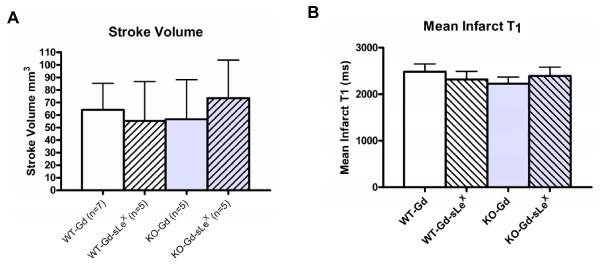
**Effects of transient cerebral ischemia on mean infarct volume and mean cerebral**. T_1_. Twenty four hours following 30 min. of middle cerebral artery occlusion infarct volumes (mean ± SD) were similar in wild-type mice (WT) or P-selectin knockout (KO) mice administered either gadolinium-DTPA (Gd) or Gd-DTPA conjugated with sialyl Lewis X (sLex) (A). Also, shown are T_1 _values in the ischemic brain prior to contrast administration. (mean ± SD) (B). Values were similar in all groups (n.s.).

All animals subjected to middle cerebral artery occlusion had ischemic infarction within the striatum and parietal cortex. Measurement of infarct size from MR images demonstrated that there was no substantial difference in the size of the stroke between the four experimental groups (figure 1A). T_1 _values, were increased in the ischemic region, however, mean values prior to contrast injection were similar between groups (Figure 1B).

The amount of P-selectin (ng/mg of protein) was determined in brain samples from brain ipsilateral and contralateral to the ischemia. Mean values were as follows: for wild type animals, 6.4 ± 5.5 in the ipsilateral cortex and 3.5 ± 1.9 in the contralateral cortex (N = 6); for P-selectin knockouts, 0.19 ± 0.3 in the ipsilateral cortex and 0.06 ± .08 in the contralateral cortex (N = 2); and for shams, 0.5 ± 0.2 in the ipsilateral cortex and 0.64 ± .09 in the contralateral cortex (N = 3). E-selectin expression was also measured in samples of brain ipsilateral and contralateral to the ischemia. Mean levels of E-selectin in ng/mg of protein were: for wild type animals, 1.048 ± 1.47 in the ipsilateral cortex and 0.789 ± 0.73 in the contralateral cortex (N = 6); for P-selectin knockout animals, 0.324 ± 0.28 in the ipsilateral cortex and 0.150 ± 0.12 in the contralateral cortex (N = 6); and, for sham animals 0.0005 ± .0001 in ipsilateral cortex and 0. 00054 ± .00002 mean contralateral cortex (N = 3). Therefore the expression of P-selectin in ipsilateral cortex of wild type animals was significantly different from ipsilateral cortex of sham animals (P < 0.001) with a trend for an ipsilateral-contralateral differences. Similarly the expression of E-selectin in ipsilateral cortex of P selectin KO animals was significantly increased compared to sham controls (P < 0.03).

### Blood Brain Barrier Function and Targeted Contrast Effects

**Figure 2:**
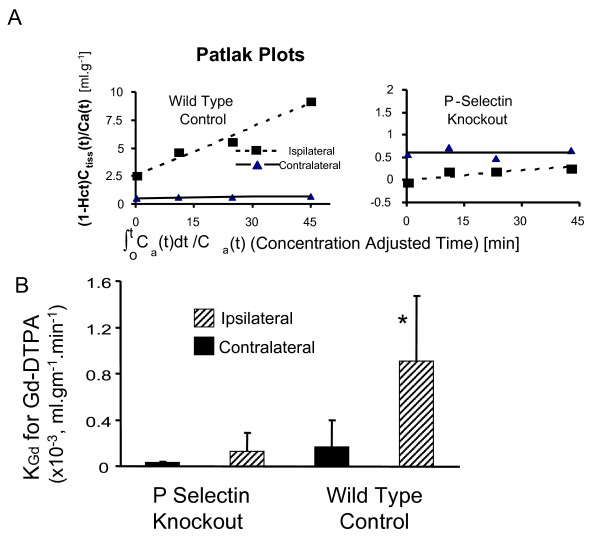
**Effect of transient focal cerebral ischemia on the transfer constant (K_Gd_) for Gd-DTPA into brain**. Shown in the upper panel (A) are the Patlak Plots used to determine K_Gd_. The y-axis in these plots is the ratio of the tissue concentration of contrast C_tiss_(t), divided by the arterial plasma concentration at that time, i.e. Ca(t)/(1-Hct), where Hct is the arterial hematocrit. The tissue concentration was determined from measures of tissue T_1 _and the blood concentration was estimated from T_1 _in the sagittal sinus. The slope provides a measure of K_Gd_. The lower Panel (B) shows that mean K_Gd _values for Gd-DTPA are less in P-selectin knockout animals compared to Wildtype controls. * P < 0.03, P-selectin Knockouts compared to Wild type groups, ipsilaterally.

Permeability of the BBB was first assessed using the transfer constant for Gd-DTPA (K_gd_) into brain of a group of wild-type mice compared to a group of P-selectin deficient mice (Figure 2). Mean K_Gd _values were greater in the ischemic cortex in wild-type mice compared to P-selectin knockouts, demonstrating enhanced BBB dysfunction in the former group.

**Figure 3:**
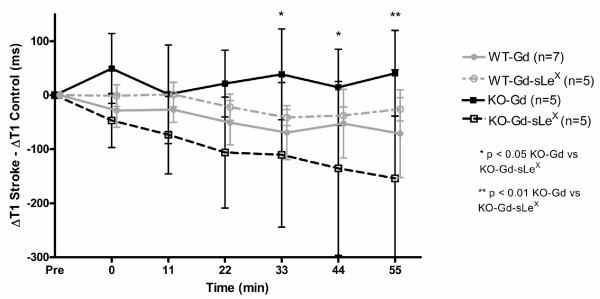
**Magnetic resonance molecular imaging with Gd-DTPA-(sLe^X^)**. In knockout (KO) mice there was increasing effects of targeted contrast Gd-DTPA-(sLe^X^) on tissue T_1 _levels in the stroke compared to the contralateral control hemisphere not observed with non-targetted contrast or Gd-DTPA. *P < 0.05 and **P < 0.01. In wild type (WT) animals the administration of Gd-DTPA-(sLe^X^) or Gd-DTPA resulted in a decrease in T_1 _in the stroke compared to contralateral control hemisphere in both groups but there no statistical difference between groups.

Selectivity of contrast accumulation or binding was also assessed by MRMI using Gd-DTPA-SleX and monitoring T_1 _changes over 55 minutes in these animals. In knockout mice, there was a significant decrease in (Δ T_1 _Stroke-Δ T_1 _Control Hemiphere) in the Gd-DTPA-(sLe^X^) group (n = 5) compared to Gd-DTPA (n = 5) at 33, 44 and 55 minutes after injection (Figure 3). No such difference could be identified in wild-type animals administered either Gd-DTPA or Gd-DTPA-(sLe^X^)). Because the Sle^X ^conjugated contrast agent binds to both E and P-selectin and P-selectin is essentially not expressed in the brain of KO mice, these results suggest there is E-selectin-mediated binding to endothelium of the conjugated contrast agent following stroke.

### Immunoglulin G or Dextran extravasation and Polymorphonuclear Leukocyte Infiltration

**Figure 4:**
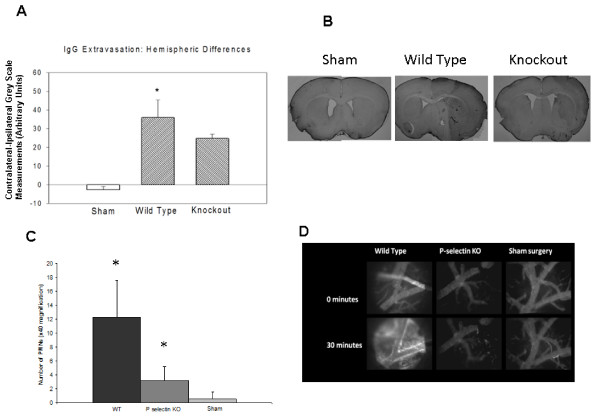
**IgG extravasation and polymorphonuclear (PMN) leukocyte infiltration after acute stroke**. The contralateral - ipsilateral difference in IgG staining was greater in wild-type than in sham surgery or P-selectin-deficient mice (A), indicating more severe BBB injury in mice expressing P-selectin (* p < 0.05 for wild-type vs. knockout (KO) mice). Also shown are representative sections stained for IgG immunohistochemistry from sham, wild type and P selectin KO mice (B). Numbers PMN leukocytes was greater in ischemic brains of wild-type animals compared to those in P-selectin deficient animals or shams (C) (* p < 0.05, vs sham). Representative intravital microscopy images of the pial vessels obtained either immediately or 30 minutes after intravenous administration of FITC-dextran (2000 kDa) in a wild-type mouse or a P-selectin knockout mouse at 24 hours after transient middle cerebral artery occlusion or a mouse subjected to sham surgery (D), FITC extravasation is evident in the wild-type mouse expressing P-selectin but not in the P-selectin KO animal.

IgG extravasation was evaluated in 4 sham surgery mice, 10 wild-type mice and 6 knockout mice (Figure 4A, B). The mean contralateral - ipsilateral differences in optical density were significantly higher in the wild-type group than in either the sham surgery or knockout groups, indicative of a more severe BBB injury in the wild-type animals (p < 0.0001).

The infiltration of polymorphonuclear (PMN) leukocytes into the brain indicated differences between groups. In the WT mice (N = 7), the number of PMNs in the ipsilateral hemisphere was significantly higher than in those of the KO (N = 6), p < 0.001) and sham mice (N = 4, p < 0.001) (Figure 4C).

Intravital microscopy was performed in 4 sham surgery mice, 9 wild-type and 5 knockout mice at 24 hours after reperfusion (Figure 4D). Fluorescein isothiocyanate (FITC)-dextran was injected and its extraluminal concentration monitored. Increased FITC fluorescence, indicative of enhanced vascular permeability, was observed by 30 minutes after FITC injection in 8/9 WT mice (89%) and in 2/5 KO mice (40%) but in none of the sham mice (0/4)(Fisher Exact 3 × 2 table, p = 0.002). The frequency of FITC extravasation for WT mice was greater than WT sham animals (Fishers Exact P < 0.01), but there was no difference in frequency between KO mice and WT sham (NS, P > 0.5). There was a strong trend for more frequent FITC extravasation observed in WT mice compared to KO mice (Fischer's Exact two tailed test P < 0.095).

## Discussion

Blood brain barrier impairment is a well-known marker of secondary cerebral injury following acute stroke associated with increases in a propensity for hemorrhagic transformation in infarcted tissue. The novel and important finding in the present study is that P-selectin expression contributes to BBB dysfunction at 24 hours after acute stroke, thus illustrating an expanded role of endothelial dysfunction as a key process in the early development of ischemic injury. In our study, early endothelial activation was evident by detecting significant cortical selectin expression whereas under normal physiologic conditions neither cerebral P- or E-selectin are detected in substantial amounts [13]. The extravasation of Gd-DTPA in the wild-type but not knockout mouse indicates that P-selectin expression is associated with more severe BBB impairment, despite similar infarct size between the two groups. Additionally, the similar T_1 _values in wild-type and knockout mice suggest comparable infarct water content and similar parenchymal injury severity [14]. Therefore, despite similar infarct extent and indicators of parenchymal injury, the E and/or P-selectin binding of Gd-DTPA-(sLe^X^) in the knockout mouse was confounded less by BBB breakdown than in the wild-type mouse, and the lack of substantial P-selectin expression in these animals supports a possible E-selectin mediated target for the Sialyl Lewis X conjugated contrast agent.

Although P-selectin expression on endothelial cell and platelet membranes has been reported to be pivotal for leukocyte recruitment during the early stroke period [1], the role of selectin adhesion molecules in promoting brain edema and BBB injury is unclear. Ruehl et al suggested that P-selectin blockade with fucoidin resulted in less brain edema at 24 hours after 4 hour middle cerebral artery occlusion [6]. In our study, there was evidence that BBB injury was more severe in the wild-type mouse as indicated by greater extravasation of Gd-DTPA and a somewhat higher frequency for extravasation of FITC than in KO mice raising the possibility of differential contributions of selectins to BBB damage. We also show that PMN infiltration into the infarct region is greater in the wild-type animals than the P-selectin knockout animals, implicating P-selectin as a major contributor to PMN recruitment and ultimately infiltration into the stroke region. Our preliminary data would also support the involvement of both P-selectin and PMN migration in early BBB breakdown. Further work will be required to elucidate the precise molecular mechanisms involved.

Previous studies have demonstrated that while ICAM-1 and E-selectin expression were necessary for neutrophil transmigration across a human brain microvessel endothelial cell layer, antibody blockade of ICAM-1 did not reduce vessel permeability to horseradish peroxidase despite dramatically decreased neutrophil migration [15]. This suggests different mechanisms involving ICAM-1 and possibly other cellular adhesion molecules underlie neutrophil migration and BBB injury. This dissociation between neutrophil-mediated cerebral injury and BBB dysfunction has also been demonstrated in traumatic brain injury[16]. While neutrophil accumulation and E-selectin expression appear to mediate blood vessel permeability to some extent after trauma, similar to our current results, mice deficient in both P-selectin and ICAM-1 had less brain edema than wild-type mice, suggesting that trauma-induced BBB injury is influenced preferentially by specific neuroinflammatory markers. Following cerebral ischemia/reperfusion, TNF-α levels are increased within two hours [17], followed by a peak in P-selectin expression at 6 hours and a later peak in E-selectin expression between 6 and 12 hours [18]. BBB breakdown after middle cerebral artery occlusion and reperfusion becomes evident at 6 hours and appears to reach its maximal extent by 24 hours [19]. The post-stroke progressive sequence of TNF-α expression, selectin upregulation and BBB injury suggests a chronological link between these events. Although many stroke patients present to emergency care at a time that is beyond the therapeutic window for thrombolysis, there remains the possibility of attenuating secondary cerebral injury in these patients by protecting the BBB. The present findings suggest a role for P-selectin as an early mediator of BBB injury and suggest an additional rationale to develop anti-inflammatory P-selectin targeted therapy to mitigate stroke-induced vascular injury.

The evidence that P selectin may be involved in increasing the size of the stroke lesion remains poorly substantiated. In our study, although we were able to demonstrate an effect of reduced BBB breakdown in P selectin knockout animals, we were not able to show an effect of reducing stroke volume. The literature that specifically addresses the issue of whether attenuating P-selectin expression reduces infarct size is sparse. Connolly et al [20] reported that isolated, purified, radiolabelled neutrophils accumulated in post-ischemic brain and that there were fewer neutrophils accumulating in P-selectin-deficient mice. In a model of cerebral ischemia reperfusion, P-selectin knockout mice exhibited a reduction in infarct volume, better functional outcome and a better return of cerebral blood flow after ischemia [20]. Similarly, blocking antibodies for P-selectin reduced infarct size and hemorrhagic transition in ischemia reperfusion [21]. The results of these studies are at least in part consistent with those observed presently regarding the beneficial effects of reduced P-selectin expression on BBB function. In moving towards clinical translation, the role for P-selectin in ischemic injury induced blood brain barrier dysfunction would need confirmation in additional animal studies that demonstrate the effectiveness on long-term and behavioural outcomes of antagonising P-selectin expression for example using either P-selectin antibody or P-selectin peptide.

## Conclusions

The results indicate that elevated P-selectin expression following ischemia-reperfusion contributes to enhanced BBB dysfunction at 24 hours after transient focal cerebral ischemia despite a lack of appreciable effect on overall infarct volume.

## Competing interests

The authors declare that they have no competing interests.

## Authors' contributions

AJ carried out the animal model, organised the data acquisition and analysis and wrote the manuscript. UT was involved in the conceptualization of the study, data analysis and acquisition, in addition to contributing to critical appraisal and writing of the manuscript. DR performed the in vivo stroke animal studies and contributed to data acquisition and analysis. JK was involved in data acquisition and tissue processing as well as statistical analysis. RM designed MR molecular contrast agent and provided critical appraisal of the manuscript. JLP was involved in the in vivo stroke studies and tissue processing as well as data analysis. SB was intimately involved in the MRI contrast agent development and the critical appraisal of the manuscript. PB conceptualized the study design, was involved in data analysis, coordinated all aspects of the study and was involved in co-authoring and critically appraising the manuscript. All authors read and approved the final manuscript.
